# Quest for Biomarkers of Treatment-Resistant Depression: Shifting the Paradigm Toward Risk

**DOI:** 10.3389/fpsyt.2013.00057

**Published:** 2013-06-18

**Authors:** Donald F. Smith

**Affiliations:** ^1^Translational Neuropsychiatry Unit, Psychiatric Hospital of Aarhus University, Risskov, Denmark

**Keywords:** depressive disorders, biomarkers, treatment resistance, immune system, cytokines, brain imaging, experimental design

## Abstract

The search for potential biomarkers of psychiatric disorders is a central topic in biological psychiatry. This review concerns published studies on potential biomarkers of treatment-resistant depression (TRD). The search for biomarkers of TRD in the bloodstream has focused on cytokines and steroids as well as brain-derived neurotropic factor. Additional approaches to identifying biomarkers of TRD have dealt with cerebrospinal fluid analysis, magnetic resonance imaging, and positron emission tomography. Some studies have also investigated potential genetic and epigenetic factors in TRD. Most studies have, however, used a *post hoc* experimental design that failed to determine the association between biomarkers and the initial risk of TRD. Particular attention in future studies should be on shifting the experimental paradigm toward procedures that can determine the risk for developing treatment resistance in untreated depressed individuals.

*“The development and use of a biomarker to identify ‘at risk’ individuals and to diagnose and/or* *quantify mental illness is the “holy grail” of psychiatry.” (Macaluso and Preskorn, 2012)*

Treatment-resistant depression (TRD) continues to challenge medical and psychological services worldwide (Rush et al., 2009; Wittchen et al., 2011; Schosser et al., 2012; Xiang et al., 2012). One way to meet these challenges would be to determine the molecular causes responsible for treatment resistance and then to develop effective treatments to help patients achieve complete remission of symptoms. That goal is, however, currently out of reach. Surely, some advances have been made toward identifying clinical risk factors for TRD along with biomarkers of affective disorders and antidepressant response (Uher et al., 2012; Perlis, 2013; Schneider and Prvulovic, 2013), but we still know next-to-nothing about risk factors for TRD (Figure 1). Our lack of information on the initial risk of TRD is, I believe, mainly due to the nature of research designs used traditionally in studies on potential biomarkers of TRD.

**Figure 1:**
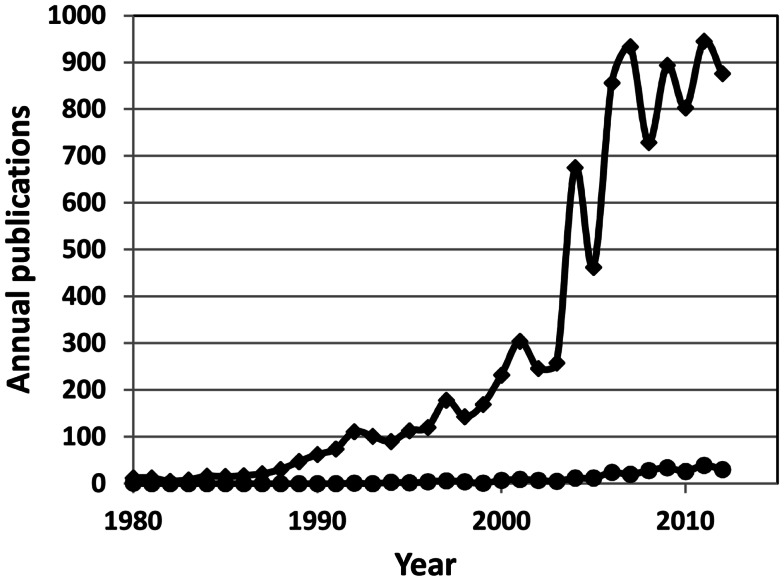
**Annual number of publications on biomarkers of human depressive disorders (diamonds) and on biomarkers of human treatment-resistant depression (circles)**.

## Research Design in Published Studies of Biomarkers in TRD

“Once upon a time, two cars collided on a bright sunny day. When a policeman arrived at the scene, he observed that a headlight was broken on one of the cars. Based on that evidence, the officer concluded that the accident was caused by a defective headlight.”

This fictitious, little tale illustrates the *post hoc*, *ergo propter hoc* fallacy (Ingle, 1972; Bowell and Kemp, 2005); it occurs when a situation observed after an event is thought to have caused the event. Errors of reasoning are surprisingly common in everyday life (Gilovich, 1993), but scientists are expected to do whatever they can to avoid them. As a result, the interpretation of findings in research projects is thought to reflect particularly critical reasoning (Martin, 1997). With regard to TRD, most studies of potential biomarkers are typically carried out in individuals that are already known to have the condition. As a result, the findings from such studies can rightfully be viewed only as the consequences of having TRD, rather than being predictive, causal, risk factors for the disorder.

## Definitions of TRD

Treatment-resistant depression is a particularly serious condition in urgent need of appropriate care. While the jury is still out concerning the definition of TRD (Ruhe et al., 2012), most studies use the criteria of an episode of major depressive disorder that has not improved after at least two adequate trials of different classes of antidepressants in a person who has complied by taking the prescribed medication (Thase and Rush, 1995; Fava, 2003; Berlim and Turecki, 2007; Berlim et al., 2008). In the author’s experience, by the time that someone with major depressive disorder fulfills those criteria, she has experienced a prolonged and extremely stressful period, characterized by intense feelings of despair, punctuated by suicidal thinking and a longing for relief from the unrelenting horrors of the disabling condition. Thus, *post hoc* biomarkers of TRD are of limited benefit for reducing the burden of disease.

## Literature Search

This review is based mainly on reports retrieved via PubMed for reports published after 1980 and identified by the following set of keywords: human depression biomarker treatment resistant. The reference lists of the retrieved articles were searched for further reports of interest. For inclusion in this review, articles must have presented a working definition of TRD and examined a potential biomarker in subjects known at some time to have the condition. No exclusion criteria were placed on the type of biomarker with regard to peripheral and central bio-molecular processes. The aim of this paper is to review the literature on biomarkers of TRD with the intention of demonstrating the necessity of shifting the experimental paradigm away from *post hoc* designs toward studies that focus on early identification of depressed individuals at risk for treatment failure.

## Peripheral Biomarkers

### Cytokines and steroids

The immune response system (IRS) and hypothalamus-pituitary-adrenal (HPA) axis have attracted attention in studies on potential biomarkers of TRD in biological psychiatry (Pariante and Miller, 2001; Rothschild, 2003; Miller et al., 2009; Dowlati et al., 2010). Bauer and coworkers published two closely related reports on immune mechanisms in patients with well-established TRD (Bauer et al., 2002, 2003). They estimated IRS activity by measuring cortisol levels in saliva samples, mitogenic proliferation capacity of lymphocytes, cytokine production in stimulated and non-stimulated and blood cells, and dexamethasone suppression of the HPA axis. The suppressant action of dexamethasone on tumor necrosis factor α (TNFα) tended to be less in TRD inpatients than in healthy subjects, whereas other biomarkers failed to differ between the two groups. Bauer and coworkers noted, however, that their findings failed to settle questions concerning predictive biomarkers of TRD. Later, O’Brien et al. (2007) measured plasma cytokine levels in depressed subjects. Their study included subjects who had failed to benefit from a single trial with a selective serotonin reuptake inhibitor (SSRI), which fails to fulfill common criteria for TRD. Plasma levels of TNFα and interleukin-6 were, nevertheless, higher in depressed subjects that were resistant to SSRI than in previously depressed, euthymic subjects that had benefited from an SSRI, but the *post hoc* experimental design cannot indicate whether plasma cytokine levels are reliable predictive biomarkers of TRD. In fact, a recent study by Raison et al. (2013) refutes the notion that high plasma levels of TNFα are involved in non-response to antidepressant drugs. They used infliximab, a monoclonal antibody which blocks TNFα, to test that notion and found no reliable relationship between experimentally induced, altered TNFα levels and antidepressant drug effects in individuals with TRD.

Markopoulou et al. (2009) investigated possible links between TRD and hyperactivity in the HPA axis. They recruited healthy subjects and inpatients with well-established TRD and measured blood levels of cortisol and dehydroepiandrosterone (DHEA). The ratio of cortisol and DHEA was used as a measure of overall glucocorticoid activity. They found that plasma levels of cortisol and cortisol/DHEA ratios were higher in TRD inpatients than in healthy subjects, but there was no correlation between the biomarkers and the clinical condition of patients. Markopoulou and coworkers cautiously concluded that the extent to which abnormalities in adrenal gland function are instrumental in the genesis of TRD remains unclear.

### Brain-derived neurotrophic factors

Brain-derived neurotrophic factors (BDNF) is the most prevalent growth factor in the central nervous system where it affects neuronal development and neuroplasticity (Sen et al., 2008; Autry and Monteggia, 2012). Fernandes et al. (2009) measured BDNF levels in serum to see whether the molecule in the peripheral circulation provides a reliable predictive biomarker for the effect of electroconvulsive therapy (ECT) in subjects with TRD. They found, however, that serum levels of BDNF failed to predict the response rate to ECT.

Anttila et al. (2007) examined the possibility that a combination of genetic polymorphisms for BDNF and serotonin type 1A (5-HT_1A_) receptors might be involved in TRD. They used blood samples to compare the genotypes of subjects with well-established TRD and healthy blood donors. They found that a particular combination of 5-HT1A and BDNF genotypes was associated with TRD. Since genetic factors are viewed as stable bio-molecular items, such findings may eventually provide biomarkers for predicting the risk of someone having TRD.

### Epigenetics

The term “epigenetics” means “outside conventional genetics.” Epigenetics is the study of stable alterations in gene expression that take place during development by random change or under the influence of the environment (Jaenisch and Bird, 2003; Tammen et al., 2013). Epigenetic processes can contribute to the presentation of depressive disorders and the effects of antidepressant treatments (Bale et al., 2010; Purcell, 2013; Tansey et al., 2013). Little is currently known, however, regarding their impact on TRD. Stewart et al. (2009) examined possible epigenetic interactions between the genotype of angiotensin converting enzyme (ACE) and antidepressant effects of ECT in subjects with well-established TRD and found no evidence for such a relationship. Shifting the paradigm to include previously untreated, depressed subjects in the analysis would expand the study of epigenetic mechanisms and the risks of the development of TRD.

## Central Biomarkers

### Cerebrospinal fluid

Monoamine oxidase (MAO) degrades biogenic amines and has been a central topic of depression research for almost 50 years (Schildkraut, 1965). In brain, molecules degraded by type A MAO (MAOA) appear in the cerebrospinal fluid (CSF). Aklillu et al. (2009) determined whether the concentration of monoamine metabolites in CSF differs between depressed subjects with or without well-established TRD. They found elevated concentrations of a dopamine metabolite, homovanillic acid, in the CSF of TRD subjects, but again, the *post hoc* design of the study failed to provide evidence on whether the biomarker can predict the risk of TRD in untreated, depressed subjects. A large-scale research project on potential biomarkers of TRD has recently been announced in which brain imaging of MAOA will take place (http://clinicaltrials.gov/ct2/show/NCT01031810); however, also this study follows a *post hoc* experimental design.

### Structural magnetic resonance imaging

Structural magnetic resonance imaging (sMRI) studies are carried out to estimate the size of brain regions. Many sMRI studies have been done in depressed subjects and healthy controls (Chen et al., 2007; Hoflich et al., 2012), but few have been directed specifically at TRD. Shah and coworkers used sMRI to see whether the size of brain regions differs between subjects with well-established TRD, subjects who had recovered from depression, and healthy subjects (Shah et al., 1998, 2002). Their initial data analysis revealed a reduction in gray matter in the left temporal cortex, including the left anterior hippocampus, in subjects with TRD compared to the other two groups (Shah et al., 1998). Some years later, the same authors did another data analysis and noted enlarged cerebroventricles and reduced gray matter in several regions of the left and right hemisphere in TRD subjects compared to the other two groups combined (Shah et al., 2002). Zhang et al. (2009) also examined brain structure in subjects who were already known to suffer from TRD. Using magnetization transfer imaging, they noted differences between TRD subjects and healthy individuals in several regions in the right hemisphere. Recently, Liu et al. (2012) also used sMRI to see whether the size of brain regions differed between patients with TRD, remitted patients, and healthy subjects. Their data analyses showed differences between groups for the size of some regions in gray matter and white matter in cerebral cortex and cerebellum. There is, however, no way of knowing whether any of these findings can provide predictive biomarkers of TRD risk, due to the *post hoc* nature of experimental paradigms.

One early study deserves special attention, because it used an appropriate experimental design for determining whether sMRI can provide predictive biomarkers of TRD. In that study, Simpson et al. (1998) examined depressed, elderly subjects at the time of their initial contact with psychiatric treatment facilities. sMRI took place as soon as possible after each subject received a diagnosis of major depression. The findings revealed a higher prevalence of white matter hyperintensities in subcortical regions in those depressed, elderly subjects that subsequently failed to benefit from at least two trials of adequate doses of two classes of antidepressant drugs and/or ECT compared with depressed, elderly subjects who benefited from the treatments. This study shows that potential biomarkers can be identified for predicting the risk of TRD by shifting the experimental paradigm away from *post hoc* designs and by moving it toward the use of baseline, pretreatment measurements of biological items, followed by regular clinical assessments and the use of objective symptom rating scales, throughout a series of carefully planned antidepressant regimes (Fava et al., 2003).

### Vascular brain disease

Naish et al. (2006) used MRI procedures to see whether pathological conditions in the cerebral microvascular contribute to TRD in subjects with late-onset disease. They measured cerebral angiopathy and the flow velocity of CSF through the cerebral aqueduct in elderly healthy subjects and depressed subjects with varying degrees of TRD. Compared to healthy subjects, patients with TRD had slower flow velocities of CSF through the cerebral aqueduct and more pronounced cerebral angiopathies, as evidenced by Virchow–Robin spaces. Naish and coworkers viewed their findings as evidence for the contribution of microvascular neuropathies in TRD, but their *post hoc* experimental design cannot distinguish between causes and consequences of prolonged and disabling depressive disorders.

### Functional magnetic resonance imaging

Li et al. (2012) used a *post hoc* design in their study of default mode functional connectivity in subjects with well-established TRD, in remitted depressed subjects, and in never-depressed healthy subjects. Under resting state functional magnetic resonance imaging (fMRI) conditions, they found that functional connectivity of brain regions was generally lower in remitted subjects and in TRD patients than in healthy subjects under resting state fMRI conditions. TRD patients had decreased functional connectivity in a network consisting of the left amygdala, anterior cingulate cortex, right insula, and precuneus cortex. Li and coworkers speculated that the particular pattern of abnormal functional connectivity in depressed subjects may constitute a biomarker of TRD, but their study design precludes statements regarding the value of resting state fMRI for identifying subjects at risk.

### Positron emission tomography

Brain imaging by positron emission tomography (PET) has often been used to study potential biomarkers of depressive disorders as well as to investigate potential beneficial effects of antidepressant drugs (Mayberg et al., 2000; Mayberg, 2007; Hoflich et al., 2012), but PET has rarely been used specifically for studying TRD. Thus, Smith et al. (2009) used the traditional *post hoc* design in their PET study of mirtazapine binding in brain regions of subjects with well-established TRD and healthy subjects. They found moderately reduced binding of radiolabeled mirtazapine in regions of the cerebral cortex and basal ganglia of TRD subjects compared with healthy subjects. Once again, the experimental design precludes statements regarding possible causal mechanisms linking reduced neuroreceptor binding and TRD.

## Concluding Remarks

Depressive disorders are complex, heterogeneous, and disabling conditions that require prompt treatment and appropriate care. Our inability to relieve the disabling symptoms of depressive disorders in many cases has stimulated research directed toward the development of more effective antidepressant treatments and improved clinical care (Rothschild, 2003; Rush et al., 2009; Brady and Insel, 2012; Schlaepfer et al., 2012). One current approach toward solving these challenges relates to research on biomarkers of depressive disorders (Frey et al., 2013).

An ultimate goal of biological psychiatry concerns the use of biomarkers to improve the initial diagnosis and treatment of depressive disorders (Cardoso de Almeida and Phillips, 2013). Unfortunately, most studies carried out in the quest for identifying biomarkers of TRD have used a *post hoc* experimental design, thereby precluding statements concerning the initial level of risk of developing treatment resistance. In the author’s view, our current lack of information on the initial risk of TRD stems mainly from the misguided post hoc experimental design of many studies that tried to identify biomarkers of TRD. To remedy this situation, the author proposes that a shift take place in the standard paradigm in biomarker studies of TRD toward examining depressed subjects at the time of their initial contact with treatment facilities. Systematic administration of antidepressant treatments with appropriate monitoring procedures in depressed individuals can be expected to identify biomarkers for predicting the risk level of treatment resistance. Such information is likely to disclose biological targets that can form the basis for developing more effective antidepressant treatments.

## Conflict of Interest Statement

The authors declare that the research was conducted in the absence of any commercial or financial relationships that could be construed as a potential conflict of interest.
